# Flocculation of kaolin particles with cationic lignin polymers

**DOI:** 10.1038/s41598-019-39135-z

**Published:** 2019-02-25

**Authors:** Agha Hasan, Pedram Fatehi

**Affiliations:** 1 Green Processes Research Centre and Chemical Engineering Department, Lakehead University, 955 Oliver Road, Thunder Bay, ON P7B5E1 Canada; 2 Key Laboratory of Paper Science and Technology of Ministry of Education, Qilu University of Technology (Shandong Academy of Sciences), Jinan, 250353 China

## Abstract

Currently, lignin of black liquor is incinerated to generate energy in pulp mills; but it has potential to be valorized through different modification methods. In this work, kraft lignin (KL) was polymerized with 2-[(methacryloyloxy) ethyl] trimethylammonium chloride (DMC) to produce cationic water soluble polymers. After producing five polymers with different molecular weights and charge densities, their flocculation efficiency in kaolin suspensions was investigated. The adsorption, zeta potential and flocculation results confirmed that the polymer with the highest charge density and molecular weight (KLD5) was a more effective flocculant than other polymers. The structure and size of flocs formed from the interaction of kaolin with KLD were determined by a focused beam reflectance measurement (FBRM). The sedimentation studies, conducted under gravitational (by vertical scan analyzer) and centrifugal force (by Lumisizer analytical centrifuge), revealed that KLD5 was very effective in flocculating kaolin particles.

## Introduction

Wastewater effluent contains a wide range of inorganic and organic matters (e.g., heavy metals, suspended particles, and aromatic molecules) imposing environmental pollutions^1,2^. Flocculation processes have been used for treating wastewater for decades. Since most naturally occurring colloids are predominantly negatively charged, the addition of cationic polymers is an effective alternative to isolate suspended particles from wastewater effluents^3,4^.

Synthetic organic polymers, such as cationic polyacrylamide (PAM) and poly diallyldimethylammonium chloride (PDADMAC), have been extensively applied as flocculants in wastewater treatment of mineral processing and papermaking operations^5–9^. These polymers were reported to form large and strong flocs with acceptable settling performance, which affords their effective removal^10^. Despite their wide range of applications, they are non-biodegradable, expensive and sometime cause health hazards^1,10^.

Recently, a considerable attention has been paid to the production of environmentally friendly polymers due to their biodegradability and renewability^11^. Natural polymers, such as starch, chitosan, and cellulose, have been widely applied as flocculants in wastewater treatments^12^. In addition, cationic polysaccharides, such as chitosan^13^, cellulose^14,15^, and starch were produced and used as flocculants in different wastewater effluents^16–18^. These polymers have been highly demanded in industrial applications, albeit their uses in food formulations. Despite the vast production of lignin as an aromatic polymer, it is still recognized as an under-utilized product of the pulping industry. In the past, the copolymerization of lignin and cationic monomers were comprehensively discussed^19–21^, but the use of polymerized lignin with (2-methacryloyloxyethyl) trimethyl ammonium chloride (DMC) as a flocculant for kaolin suspension has not been reported, which was the first objective of this work.

When flocculants adsorb on particles, they change the physicochemical properties of the particles, which leads to their agglomeration following altered mechanisms^7,8,22^. Numerous investigations have demonstrated that the flocs formed through bridging of particles can be settled readily^23–27^, while flocs formed via charge neutralization and patching are more difficult to sediment^7,23,28,29^. As it is unclear how lignin-based polymers would agglomerate particles, the second purpose of this paper was to investigate how the charge density and molecular weight of lignin-DMC polymers impact the formation and settling of agglomerated flocs.

The kaolin suspension has been used as a representative of colloidal systems for decades because its surface characteristics are well-understood to allow investigations on flocculation and sedimentation processes^30–33^. The surface of kaolin particles is heterogeneous consisting of positive charges at the edges and negative charges on the basal face, and the interaction of these charges with surrounding environment promotes the aggregation of particles^34^. The results generated in this work will facilitate the analysis on the actual wastewater effluents, which is the objective of our future work.

In this study, kraft lignin-based polymers were produced via free radical polymerization of DMC and lignin. The flocculation behavior of the polymers (KLD) with different molecular weights and charge densities was investigated in a kaolin suspension for the first time. This paper presents the correlations developed between the properties of lignin-based polymers and their flocculation performance and settlement under different conditions using advanced tools. The objective of this work was to investigate how the charge density and molecular weight of lignin-DMC polymers would impact its flocculation performance. However, the flocculation mechanism and its impact on settling the agglomerated flocs will be studied in our future work.

## Results and Discussion

### Polymerization

The polymerization of kraft lignin and DMC was performed following a free radicle polymerization mechanism as explained in our previous work^35^. In the reaction mixture, potassium per sulfate generated two sulfite radical anions by thermal decomposition (Figure S1a). The sulfite radicals attacked the hydroxyl group (OH) of KL to form phenoxy radicles. These phenoxy radicals then reacted with double bonds of DMC to have them engaged in the polymerization reaction to produce KLD polymers (Figure S1b). On the other hand, sulfate radicals could initiate the homopolymerization of DMC to produce homopolymers (PDMC) as a by-product (Figure S1c).

### Properties of KLD

The reaction conditions and properties of KLD polymers produced via free radical polymerization of KL and DMC are listed in Table 1. The phenolic hydroxyl group and carboxylate group contents of KL were 1.72 mmol/g and 1.01 mmol/g, respectively^36^. Under the experimental conditions studied, the polymers with varying charge densities and molecular weights were synthesized. It is evident that the nitrogen content, charge density, and molecular weight increased, confirming the grafting of DMC onto lignin backbone^37^. The grafting ratio of DMC to lignin ranged from 17% to 198.7% for the KLD polymers. The results also indicated that varied DMC amounts were responsible for different nitrogen contents, charge densities, and molecular weights of KLD polymers, which were closely related to their flocculation performance. In addition, the cationic charge densities of five KLDs were theoretically calculated from their nitrogen content, since 1 mol of quaternary ammonium group contained 1 meq charge density. The theoretical charge densities were close to experimental values listed in Table 1. The reaction of KL and DMC was described in our previous work^35^.

**Table 1:** Reaction conditions and properties of KL and KLDs.

Conditions	KL	KLD1	KLD2	KLD3	KLD4	KLD5
DMC, mol	—	0.009	0.009	0.014	0.019	0.024
KL, mol	—	0.027	0.016	0.011	0.011	0.022
pH	—	3	4	5	3	4
Temperature, °C	—	80	90	90	70	80
Time, h	—	4	5	4	5	3
Experimental charge density, meq/g	−0.2	0.74	1.52	2.5	2.93	3.66
Theoretical charge density, meq/g	0.021	0.70	1.47	2.41	2.86	3.60
Nitrogen, wt.%	0.03	0.98	2.05	3.40	3.97	5.05
Grafting ratio, wt.%	—	17.0	43.7	101.8	143.3	298.7
Solubility, wt.%	5	39	40	42	56	66
M_w_, g/mol	17,800	30,300	50,700	81,900	109,100	162,600
M_n_, g/mol	5,100	22,700	35,400	56,200	70,600	96,200
M_w_/M_n_	3.49	1.33	1.43	1.46	1.54	1.69

### Adsorption of KLD on kaolin

**Figure 1:**
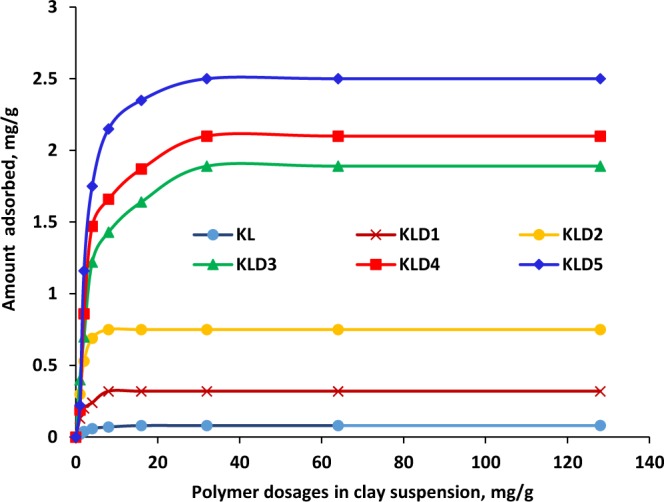
Adsorption of KL and KLD on kaolin particle as a function of polymer dosage, conducted under the conditions of pH 7, 25 °C, 1 h and 0.4 g/L clay concentration.

Figure 1 shows the adsorption behavior of KL or KLD on the surface of kaolin particles as a function of polymer dosage at pH 7. The adsorption increased as the charge density and molecular weight raised. This phenomenon may be attributed to electrostatic interaction between cationic KLD and negatively charged clay particles^38^. Previous studies demonstrated that the adsorption of cationic polymers increased with increasing their charge density^39,40^. Wang and coworkers^37^ articulated that an increase in the charge density of cationic xylan from 1.8 to 2.4 meq/g enhanced the adsorption of cationic polymer from 3.1 to 4.6 mg/g onto kaolin particles at a 16 mg/L of polymer dosage. In another study, with increasing the concentration of cationic starch from 30 to 260 g/L in a clay suspension, the adsorption of cationic starch on the clay surface increased from 2 to 18 mg/g^41^. KL had a limited adsorption on kaolin particles.

### Dynamic flocculation

**Figure 2:**
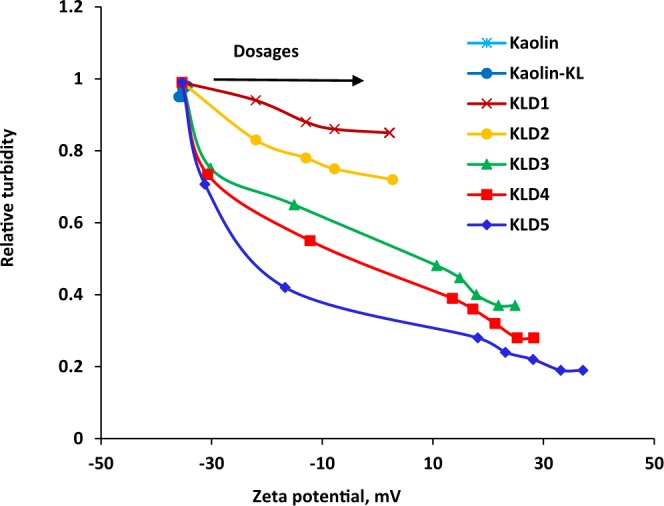
Effect of zeta potential on the relative turbidity of kaolin suspensions, conducted under the conditions of pH 7, 1 h, 25 °C, and 0.4 g/L of kaolin concentration.

Figure 2 demonstrates the relative turbidity of kaolin suspensions as a function of their zeta potential. It is seen that, by raising the dosage of KLD, the zeta potential became more positive, and the maximum zeta was obtained for KLD5. Also, the relative turbidity of the suspension was reduced as the dosage increased, and the minimum relative turbidity was obtained for KLD5. It is noticed that when KLD1 and KLD2 were added to the clay suspension, the relative turbidity of the suspension was gradually decreased, and their zeta potential became close to neutrality. These results may provide evidence that the flocculation probably occurred via charge neutralization mechanism. For KLD3, KLD4 and KLD5, the relative turbidity of the suspension decreased when the zeta potential of kaolin particles became more positive, indicating that both charge neutralization and bridging mechanisms probably involved in the flocculation process. Interestingly, the results confirmed that the charge density and molecular weight of KLD are critical factors in altering the zeta potential and relative turbidity of the suspension, and these changes are attributed to the adsorption of KLDs on clay particles.

**Figure 3:**
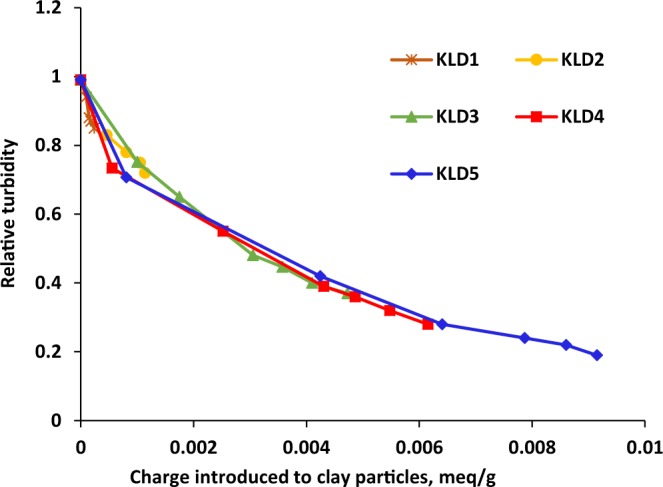
Effect of total charges introduced to particles on the relative turbidity of kaolin suspensions, conducted under the conditions of pH 7, 1 h, 25 °C and 0.4 g/L of clay concentration.

Based on the results obtained from the adsorption analysis (Fig. 1), and the charge density of KLDs, it was possible to calculate the total charges introduced on the surface of kaolin particles after KLD adsorption. Figure 3 showed the influence of charges, which was introduced to the particles via adsorbing KLD, on the relative turbidity of the suspensions. As observed, KLD5 introduced more charges onto kaolin particles as compared to the other KLDs, and hence they reduced the relative turbidity more significantly (Fig. 3). These findings confirmed that the relative turbidity of clay suspension was strongly affected by the amount of KLD adsorbed and the total charges introduced on kaolin particles. Among the cationic polyacrylamides that Ariffin and coworkers^42^ studied in the flocculation of palm oil mill effluent, the flocculant with the molecular weight of 1.5 × 10^6^ g/mol was the most effective one in turbidity removal.

### Floc size measurement

**Figure 4:**
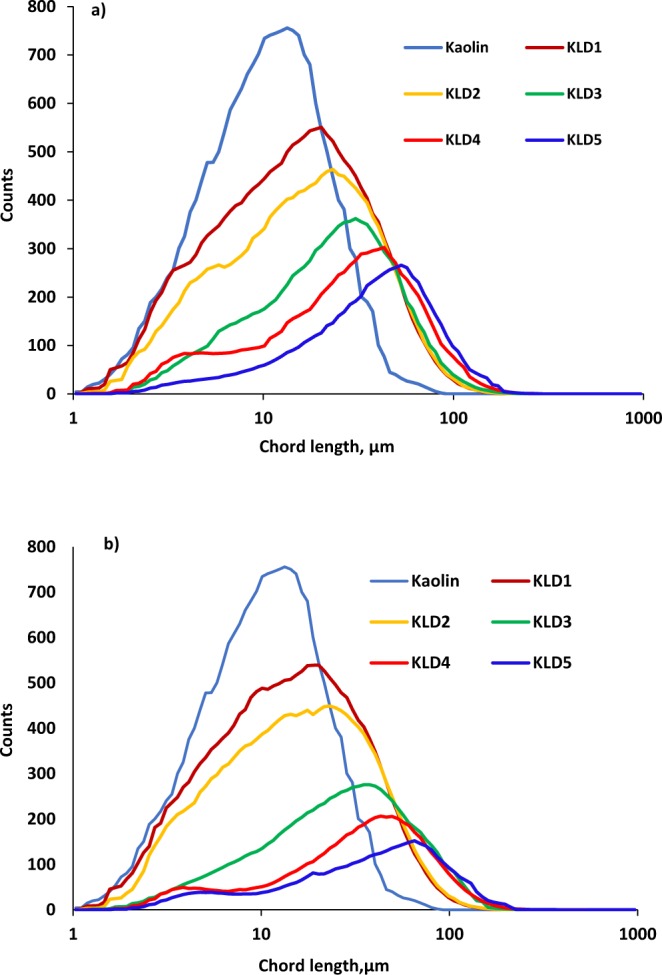
Chord length distribution of flocs formed at dosage of (**a**) 8 mg/g and (**b**) 32 mg/g conducted under the conditions of pH 7, 25 °C and 0.4 g/L of kaolin concentration.

The size distribution of flocculated kaolin particles was shown in Fig. 4 for two different dosages of 8 and 32 mg/g KLD in clay suspension. The addition of KLD to the kaolin suspension widened the size distribution of particles in the suspension, while reducing their counts. These results were in conformity with those discovered by Thapa *et al*.^43^, where high molecular weight and charge density polyacrylamide polymers were more effective than the low charged and low molecular weight ones in reducing the particle’s counts in sludge flocculation process. The results also confirmed that KLD5 widened the size distribution (and reduced the counts of flocs) more greatly than did other KLDs. The dosage of KLDs did not change the size of the flocs formed via treating with KLD1 and KLD2, but other KLDs increased the chord length at a higher dosage. In particular, the average chord length of KLD5 was 53 µm at 8 mg/g dosage, while that was 65 µm at 32 mg/g dosage. These results confirmed that higher molecular weight KLDs enhanced the flocculation efficiency and thus aggregation of the clay suspension^44,45^.

### Flocculation of kaolin under non-stirring conditions

**Figure 5:**
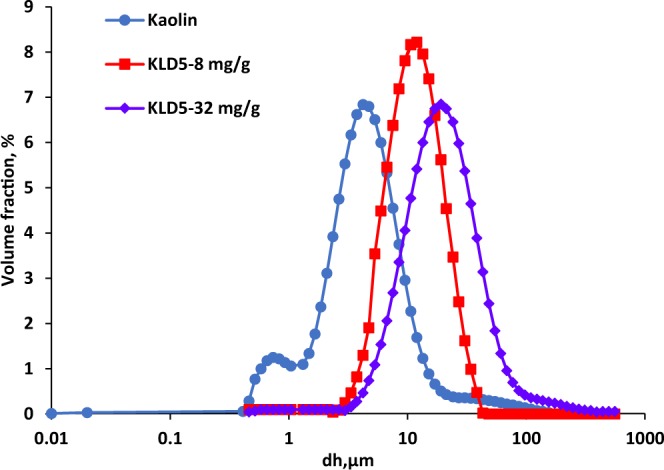
The floc size distribution of kaolin particles in the presence and absence of KLD at dosage of 8 and 32 mg/g, conducted under the conditions of pH 7, 25 °C and 0.4 g/L of kaolin concentration.

As KLD5 was more effective than other KLD, the analysis on non-stirring conditions was performed using only this polymer. The volume fraction of flocs in the suspension was plotted as a function of their hydrodynamic diameter (d_h_) in Fig. 5. As observed, the flocculation of kaolin particles by KLD increased the average size of flocs. The particle size of kaolin was 4.7 µm without using polymers. The floc size of KLD5 grew to 16.85 and 19.12 µm at 8 and 32 mg/g dosages, respectively. These results are consistent with those presented in Fig. 4. It was claimed that the floc size would increase by raising the polymer’s charge density and molecular weight^39^. In addition, these results indicated that increasing the KLDs dosage enhanced the floc growth as well^46^.

**Figure 6:**
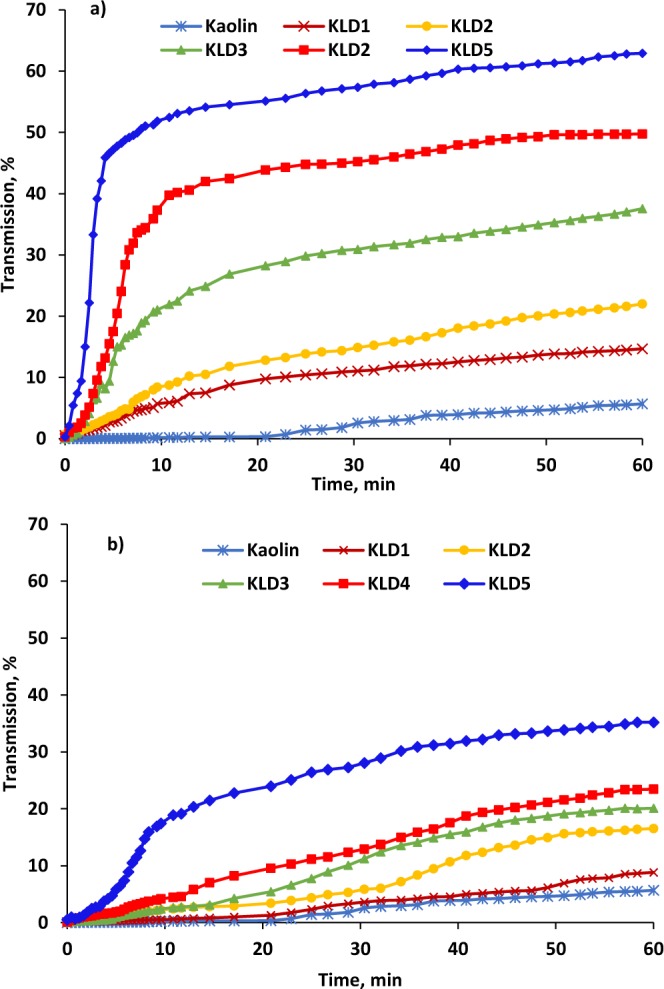
Transmission intensity of kaolin suspension (top layer) at the dosage of (**a**) 8 mg/g and (**b**) 32 mg/g, as a function of time, conducted under the conditions of 0.4 g/L clay at pH 7, 25 °C and 1 h.

The impact of KLD on the transmission (%) of kaolin suspensions is shown in Fig. 6. In the absence of KLD, the transmission of kaolin particles was the lowest (5.67%) of all studied systems. After adding KLD, the particles in the kaolin sample started to settle, which made a clear and transparent layer on top of the sample, and this increased the transmission of the samples. The increase in the transmission was more obvious for 32 mg/g dosage than for 8 mg/g dosage (Fig. 6). For example, the addition of KLD5 exhibited large sedimentation with fastest transmission increase to 35.20% and 62.91% at 8 and 32 mg/g of dosages, respectively. This improved settling behavior of kaolin suspension may largely be attributed to the higher adsorption of KLD5 onto clay particles as well as its higher charge density and molecular weight (Fig. 1).

The settling velocity and the compactness of sediments after 1 h of settling in the absence and presence of KLD are listed in Table 2. The settling velocity is inversely related to the compactness of KLD. The smaller the settling velocity, the larger the compactness would be. Kaolin particles had the highest sediment compactness (42.76 g/L) and the lowest settling velocity (15.3 mm/h). At a 8 mg/g dosage, the sediment compactness decreased from 42.76 g/L to 37.73 and 14.18 g/L; whereas, the settling velocity increased from 17.2 to 130.7 mm/h for KLD1 and KLD5, respectively. A similar trend was observed for 32 mg/g dosage, but generally the compactness was smaller and settling velocity was faster. These results confirmed that the compactness of the settled flocs decreased with increasing the molecular weight of KLD. High molecular weight KLDs bridged the kaolin particles to form large porous flocs containing water that reduced its compactness. The formation of loose flocs via bridging mechanism is well documented in the flocculation of cement, kaolin, and hematite in the past^6,47,48^. The faster settling velocity of KLD5 (173.4 mm/h) confirmed that the flocs of higher molecular weight KLD were larger than those of lower molecular weight ones (Figs 4 and 5)^6^.

**Table 2:** Settling velocity and compactness of settled flocs after settling in 1 h of experiment.

	Blank	KLD1		KLD2		KLD3		KLD4		KLD5	
Dosage (mg/g)		8	32	8	32	8	32	8	32	8	32
Sediment compactness (g/L)	42.76	37.73	35.01	33.99	30.78	26.19	20.59	18.15	13.93	14.18	10.85
Settling velocity (mm/h)	15.3	17.2	18.6	32.4	37.4	53.3	75.2	88.4	120.5	130.7	173.4

### Size of suspended flocs

**Figure 7:**
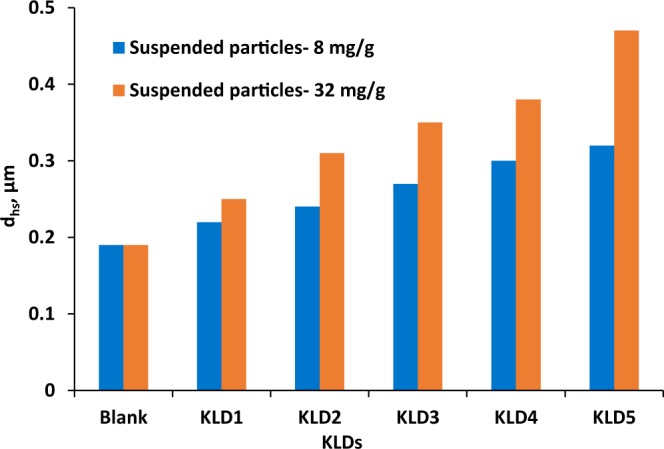
The size of suspended particles in the presence and absence of KLD conducted under the conditions of 0.4 g/L clay at pH 7, 25 °C and 1 h.

The hydrodynamic diameter of suspended kaolin particles was determined in the absence and presence of KLD in Fig. 7. The d_h,s_ was approximately 0.19 µm for the suspended kaolin particles. For the samples treated with KLDs, the d_h,s_ was insignificantly different from that of kaolin particles, indicating that the size of suspended particles did not remarkably change after treating with KLD, and the formed large flocs were probably settled. On the other hand, there was a slight increase in the size of the suspended particles as the size of KLD increased in the system. These outcomes comply with the experimental results presented in Fig. 5. As these larger flocs were loosely bound with KLD, they had a relatively low compactness, which led to their suspension along with smaller flocs.

### Sedimentation behavior under accelerated gravitation

**Figure 8:**
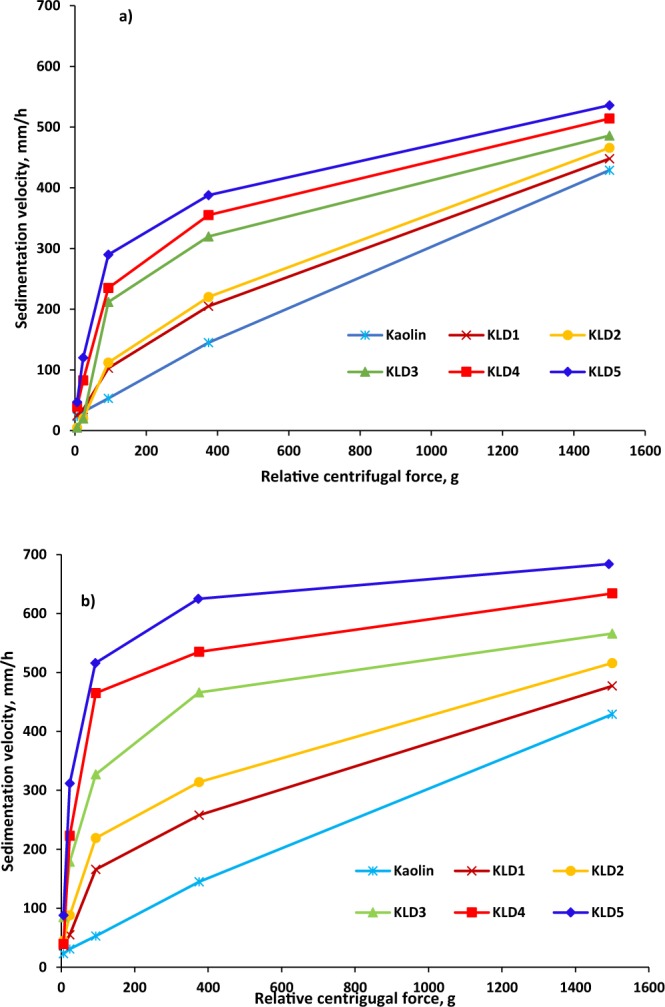
Sedimentation velocity of the KLD in kaolin suspension at the dosage of (**a**) 8 mg/g and (**b**) 32 mg/g as a function of RCF values conducted under the conditions of 0.4 g/L clay at 25 °C, pH 7 and 5 min.

The sedimentation velocities of KLDs at various RCFs are summarized in Fig. 8 for two dosages of 8 and 32 mg/g KLD in kaolin suspension. Generally, flocculants agglomerated particles and thus there was a change from a monodisperse to polydisperse system^49^. As can be seen, the trend of sedimentation velocity was similar for all of the KLD at 8 mg/g and 32 mg/g dosages, but a more dramatic increase in sedimentation velocity was observed for the KLD5 than other KLDs with accelerating centrifugation. These results are in agreement with the results obtained in Table 2. The higher settling rate of KLD-induced flocs could be due to their orientation in parallel to the centrifugal direction at a high RCF^31,50^. Because of the polydispersity of the flocs, the flow resistance of KLD/kaolin particles was different from that of a spherical particle. Thus, the drag force experienced by the flocs was smaller and the settling velocity became faster when flocs were oriented parallel to the flow field. Similar phenomena were reported by Chang and Liao^51^ in measuring the sedimentation velocities of titanium oxide (TiO_2_) nanoparticle under centrifugal forces. These results indicated that the KLD made flocs in the suspension did not settle uniformly under altered centrifugal forces. This study also confirmed that the rate of floc sedimentation in a kaolin suspension mainly depended on the particle size and density.

**Figure 9:**
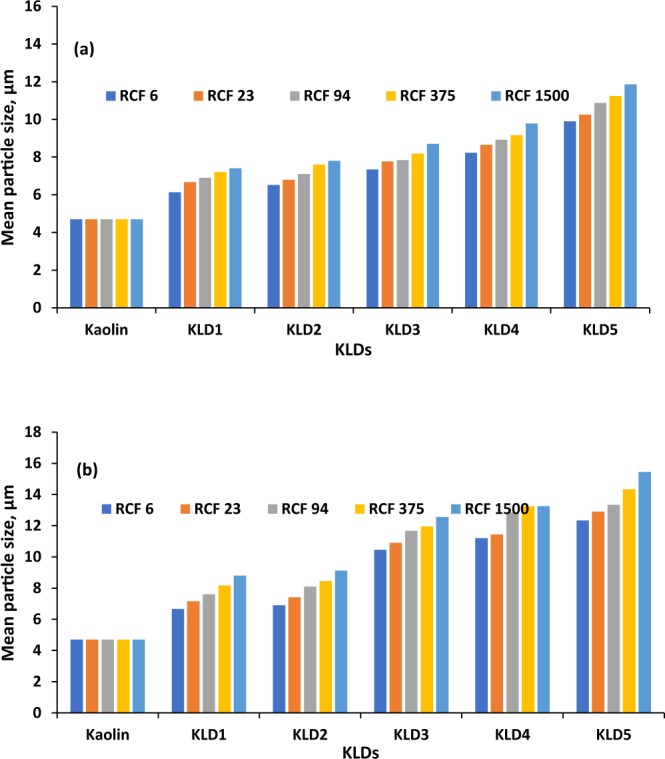
Particle size distributions obtained via centrifugation analysis in the presence or absence of KLDs at the dosage of (**a**) 8 mg/g (**b**) 32 mg/g as a function of RCF values; under the conditions of 0.4 g/L clay at 25 °C, pH 7, and 20 min.

The volume-weighted particle size distribution of the flocs was identified by analyzing the time evolution of the transmission at a fixed position and the results are shown in Fig. 9. In the absence of KLD, kaolin particle size in stable dispersion was 4.7 µm. The addition of KLD led to the formation of larger particles that could settle faster, which are in good agreement with data reported in Figs 5 and 8. As observed, the particle size increased from KLD1 to KLD5 by increasing the centrifugal force. Claverie and coworkers^52^ reported that the particle size of silica dispersions increased with polymer concentration as well as polymer molecular weight.

### Conclusions

The efficiency of KLD5 was higher than other KLDs in flocculating kaolin particles due to its higher adsorption as well as its charge density and molecular weight. The relative turbidity of the suspension dropped more quickly, and its zeta potential increased more dramatically with KLD5 than other KLD as it adsorbed more than other KLDs on kaolin particles. The sedimentation studies under gravitational force demonstrated that the compactness of KLD flocs decreased, while the settling velocity increased progressively with increasing concentration as well as the charge density and molecular weight of KLD. By increasing the centrifugal force, the size of the flocs and the settling velocity were increased regardless of the KLD properties. Also, the sedimentation velocities of KLD5 induced flocs were 184 mm/h and 244 mm/h, while its floc size increased to 10.98 µm and 13.69 µm at 8 and 32 mg/g of dosages, respectively. Addition of KLD resulted in change from monodisperse to polydisperse settling due to agglomeration/flocculation of kaolin particles by KLD.

## Materials and Methods

### Materials

Softwood kraft lignin was generated via LignoForce^TM^ technology at FPInnovations’ Thunder Bay (ON) facility^53^. 2-[(methacryloyloxy) ethyl] trimethylammonium chloride (DMC) (80% in water), potassium persulfate (K_2_S_2_O_8_) (analytical grades) and kaolin were supplied by Sigma-Aldrich. Polydiallyldimethylammonium chloride (PDADMAC) with the molecular weight (M_w_) of 100,000–200,000 g/mol was purchased from Sigma Aldrich. Potassium polyvinyl sulfate (PVSK) with a M_w_ of 100,000–200,000 g/mol (97.7% esterified) was purchased from Wako Pure Chem. Ltd. Japan. All chemicals were applied without further purification. Moreover, ethanol (95 vol. %) was received from Fisher Scientific company. Silicon oil and tetrafluoroethylene were received from formulation and used as standard chemicals for transmission and backscattering detectors of a vertical scan analyzer, respectively.

### Lignin-DMC production and purification

Kraft lignin-DMC polymers were synthesized in 250 mL three-neck glass flasks under the reaction conditions illustrated in Table 1. After the reactions, the flasks were submerged in cold tap water for 20 min. Then, ethanol (80 vol. % in water) was mixed with the reaction media to precipitate the lignin-based polymers from the systems^35,37^. After a 10-min centrifugation at 3500 rpm, the precipitated copolymer (KL-DMC) was collected and the homopolymer (PDMC) and unreacted monomers (DMC) present in the supernatant were removed from the suspensions. The collected precipitates were then mixed with ethanol (80 vol.%) for 5 min. The mixtures were centrifuged again, and this process was repeated 3 times, where the final product is considered lignin polymer, KLD. Subsequently, the precipitated KLD polymers were mixed with 200 mL of deionized water, while adjusting the pH of the solution to 7.0 ± 0.2 prior to use. The solubility, charge density, molecular weight and elemental components of KLD were determined as explained in our previous work^37,54^.

### Particle size analysis

The hydrodynamic diameter of kaolin particles and flocs of KLD/kaolin was measured by a particle size analyzer, MasterSizer 2000 (Malvern Instruments). In this study, 1 g of clay suspension (20 g/L) was mixed with 50 mL of distilled water or 50 mL of KL or KLD solution so that KL or KLD dosage in the system remained at 8 and 32 mg/g dosages (based on kaolin). The system was stirred at 300 rpm and room temperature for 2 h to make the samples ready for particle size analysis. The measurement was conducted at the wavelength of 633 nm with red laser light. The mean value of hydrodynamic size, d_h_, of the samples was determined as the average median value of three parallel measurements.

### Adsorption studies

For studying the adsorption of KL or KLD on clay particles, KL or KLD was mixed with 50 mL of kaolin (clay) suspensions (0.4 g/L) to make 1 to 128 mg/g dosage of KL or KLD in clay suspensions. The suspensions were stirred at 300 rpm for 1 h at room temperature. Afterward, the suspensions were centrifuged for 15 min at 3500 rpm and then the concentrations of the KLD remained in the supernatants were determined by a UV/Vis spectrophotometer (Genesys 10 S UV/vis, Thermo FisherScientific, USA) at the wavelength of 205 nm. The impact of pH was also studied on the adsorption of KLD on clay particles. The pH of the kaolin suspensions (ranging 2 to 12) was adjusted with 0.1 M NaOH solution or H_2_SO_4_ prior to adsorption experiments, and the steps indicated above were followed accordingly.

### Zeta potential analysis

A NanoBrook Zeta PALS (Brookhaven Instruments Corp, USA) was used for zeta potential analysis of clay suspensions. The pH adjustment of the suspensions was carried out using 0.1 M NaOH or H_2_SO_4_. The clay samples (1 g at 20 g/L concentration) with or without the polymers were mixed with 50 mL of distilled water and stirred under the same conditions stated in the adsorption studies section, then their zeta potential was measured in a 1.0 m M KCl^28^. All the measurements were carried out at room temperature at a constant electric field (8.4 V/cm).

### Flocculation analysis

The relative turbidity of the suspension was determined using a photometric dispersion analyzer (PDA 3000, Rank Brothers Ltd) that was attached to a dynamic drainage jar (DDJ) fitted with a 200-mesh screen^55,56^. Herein, 500 mL of distilled water was first added to the DDJ and transported from the DDJ to the PDA through a 3 mm plastic tube (Tygon, R-3603) until a steady flow rate of 20 mL/min was achieved. Then, 10 mL of a 20 g/L clay suspension was added into the DDJ at 300 rpm. The relative turbidity of the suspensions containing polymers was investigated as explained by Wang *et al*.^37^.

### Focused beam reflectance measurement

The change in the size of kaolin and KLD/kaolin flocs was assessed in a real-time scenario by a focused beam reflectance measurement (FBRM, Mettler-Toledo E25). The chord length distribution of particles in the suspension was determined over the range of 1 and 1000 µm using IC-FBRM software^57^. 500 mL of distilled water was mixed with 10 mL of a 20 g/L clay suspension at 300 rpm. This experiment was repeated for the suspensions containing KLD with the dosages of 8 mg/g and 32 mg/g (based on dried weight of clay particles)^37^.

### Gravitational sedimentation analysis

The sedimentation performance of kaolin particles under non-stirring conditions in the absence or presence of KLD were assessed by a vertical scan analyzer, Turbiscan (Lab Expert, Formulaction). In this analysis, different dosages of KLD were added to the kaolin suspension. After stirring at 300 rpm for 2 min, 20 mL of suspensions were added to the cylindrical glass cells for further analysis. Electro luminescent diode light at 880 nm scanned the cell from bottom to top at a 40 µm height interval. The scanning process was conducted every 25 s and the experiment lasted for 1 h^58^.

Particles of different sizes produced by KLD would settle at different rates when flocculated. This settlement would clear the top part of the suspension. The variations in transmission and backscattering data, collected from the top and bottom parts of the sample after settling for 1 h, were considered for evaluating the efficiency of KLD in flocculating kaolin suspensions.

The hydrodynamic diameter of the suspended particles in the system (d_h,s_) was determined by the transmission data from the top layer and the mean volume fraction of particles after settling for 1 h based on Lambert-Beer law (equations (1) and (2)).

1 $$T(l,{r}_{i})={T}_{0}{e}^{\frac{-2{r}_{i}}{l}}$$

2 $$l({d}_{h,s},{\varphi }_{s})=\frac{2{d}_{h,s}}{3{\varphi }_{s}{Q}_{s}}$$

where r_i_ represents the internal radius of the measurement cell, l is photo mean free path, T_0_ is the transmittance of continuous phase (i.e., water), and T is the transmittance of suspension (i.e., kaolin suspension)^58,59^. Therefore, the transmission data collected by the instrument directly depended on the mean hydrodynamic diameter of the suspended particles, d_h,s_, and their volume fraction, ϕ_s_. This study helped develop a correlation between particles’ diameter and their volume fractions in the solution.

The compactness of the sediment was determined as the ratio of the mass to volume for the settled flocs after 1 h of settling. For this purpose, samples were collected (and dried at 105 °C overnight) from the top part of the suspension before and after 1 h of treating the suspensions with KLD. In this analysis, the settling velocity of the flocs was determined as the rate of sediment thickness grows with respect to time^60^.

### Centrifugal sedimentation analysis

Sedimentation velocities of kaolin and KLD/kaolin flocs were measured by an analytical centrifugation analyzer, Lumisizer (LUM GmbH, Germany)^61^. In each measurement, 1 mL of a 0.4 g/L clay suspension was transferred into the cell of the instrument, centrifuged at different revolutions (200, 400, 800, 1600, and 3200 rpm) for 20 min, and the transmission data was collected after every 5 s. Measurements were performed at 25 °C and a light factor of 1. This experiment was conducted for the suspensions containing KLD/kaolin at the dosages of 8 mg/g and 32 mg/g based on dried weight of clay particles in the suspensions. Kaolin sample (0.4 g/L) was also used as a blank (without polymer). The relative centrifugal force (RCF) was calculated using equation (3), where r is the radius of centrifuge in mm.

3 $${\rm{RCF}}={(\frac{RPM}{1000})}^{2}r\times 1.18$$

For each run at different RCF (6, 23, 94,375,1500 × g), the thickness of sedimentation layer in the cell was plotted as a function of time, the slope of which led to the sedimentation velocity (mm/h) of the particles at an RCF. By plotting the sedimentation velocity against RCF, an estimation of settling rate of KLD was identified.

Under the aforementioned experimental conditions, the size distribution of flocs was determined. The volume-average diameters were measured by constant position analysis^62^. In each measurement, three positions (approximately 115.0 120.0, 125.0 mm) within the detection region (105–130 mm) were chosen to ensure that the results of the analysis were representative for all measurements.

## Supplementary information

Supplementary Materials
