# Supplementary material for: Flocculation of kaolin particles with cationic lignin polymers
Source: Sci Rep. 2019 Feb 25;9:2672. doi: 10.1038/s41598-019-39135-z (PMC6389989; doi:10.1038/s41598-019-39135-z)
Supplement: Supplementary file 1 — Supplementary Materials [file 41598_2019_39135_MOESM1_ESM.docx]

**Flocculation of kaolin particles with cationic lignin polymers**

Agha Hasan^1^, Pedram Fatehi*^1,2^

^1^Green Processes Research Centre and Chemical Engineering, Department, Lakehead University, 955 Oliver Road, Thunder Bay, ON, Canada, P7B5E1

^2^Key Laboratory of Paper Science and Technology of Ministry of Education, Qilu University of Technology (Shandong Academy of Sciences), Jinan, China, 250353

*corresponding author, email: [pfatehi@lakeheadu.ca](mailto:pfatehi@lakeheadu.ca); tel: 807-343-697; fax: 807-346-7943

**
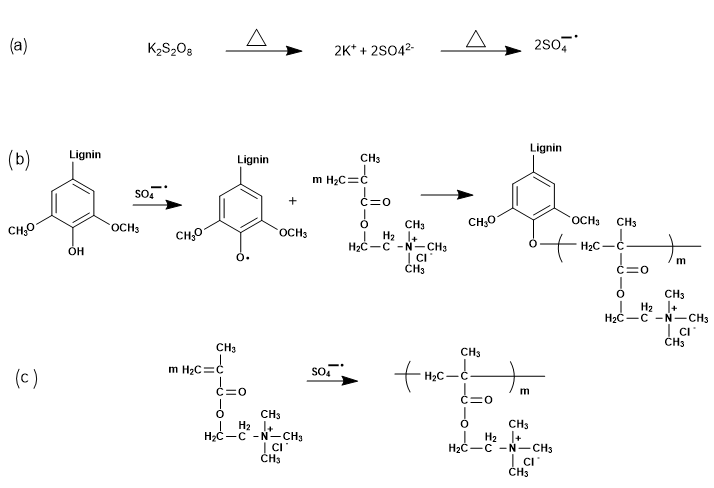
**

Figure S1. Mechanism of the polymerization reaction of KL and DMC (a) thermal decomposition of potassium per sulfate, (b) polymers of KLD, and (c) homopolymers of PDMC.
